# Twenty-five-year trends in breastfeeding initiation: The effects of sociodemographic changes in Great Britain, 1985-2010

**DOI:** 10.1371/journal.pone.0210838

**Published:** 2019-01-17

**Authors:** Deon A. Simpson, Maria A. Quigley, Jennifer J. Kurinczuk, Claire Carson

**Affiliations:** National Perinatal Epidemiology Unit, Nuffield Department of Population Health, University of Oxford, Oxford, United Kingdom; Christiana Care/University of Delaware, UNITED STATES

## Abstract

**Background:**

Data from the UK Infant Feeding Surveys indicate that breastfeeding initiation increased between 1985 and 2010. During this period, societal changes in GB also influenced the sociodemographic characteristics of women in the childbearing population. As breastfeeding behaviour is highly socially patterned in GB, the increasing trend in breastfeeding initiation may have hidden inequalities in breastfeeding practices. This study examines the sociodemographic inequalities in breastfeeding initiation in GB between 1985 and 2010, exploring whether and how this may have been influenced by social and policy changes.

**Methods:**

Data drawn from the nationally representative 1985, 1990, 1995, 2000, 2005 and 2010 Infant Feeding Surveys were used to estimate changes in the proportion of mothers in selected sociodemographic groups over time. Logistic regression models estimated the independent associations between breastfeeding initiation in each survey year and maternal sociodemographic characteristics. Associations were adjusted for maternal sociodemographic, pregnancy-related and support factors. Evidence of a change in the association between breastfeeding initiation and each sociodemographic characteristic over time was assessed using a test for statistical heterogeneity.

**Results:**

The sociodemographic characteristics of mothers in GB changed substantially between 1985 and 2010. Mothers were increasingly more likely to be 30 or over; have higher education and socioeconomic status; and be single or cohabiting. An increasing proportion of mothers in GB identified as being of black or minority ethnic origin. Reported smoking in pregnancy declined. These same characteristics independently predicted higher odds of breastfeeding initiation; the associations between these characteristics and breastfeeding initiation did not vary significantly over time.

**Conclusions:**

Marked inequalities in breastfeeding initiation persisted over the study period, hidden among the increasing initiation rate at the population level. The increasing overall rate of initiation was most likely driven by the rising prevalence of those groups of mothers who were, and remain, characteristically most likely to breastfeed.

## Introduction

The World Health Organisation recommends exclusive breastfeeding for the first 6 months after birth, followed by continued breastfeeding alongside safe and appropriate foods until at least 2 years [1]. Despite similar recommendations in Great Britain (GB) [2, 3], breastfeeding rates remain relatively low compared to rates in other high-income countries [4]. From 1975 to 2010, a quinquennial national survey, the Infant Feeding Survey (IFS), was used to monitor how thousands of recent mothers in GB feed their baby in the first 8–10 months. Available data from the 1985 to 2010 IFS suggest that breastfeeding rates rose steadily during this period. Between 1985 and 2010, the rate of breastfeeding initiation increased from 64% to 81%, and the rate of breastfeeding continuation at six months among all mothers rose from 21% to 34% [5]. Although these increasing trends are encouraging, evidence from several studies suggests the presence of sociodemographic disparities in the practise of breastfeeding in GB. In particular, breastfeeding initiation and continuation rates are highest among mothers who have higher education; who are older; who are married or living with a partner; from higher socioeconomic groups; and who are of black and minority ethnicity [6–11]. Therefore, it is possible that the increasing breastfeeding rates from 1985 to 2010 masked inequalities in breastfeeding practices between groups of mothers. Consequently, in each survey year from 1985 to 2010, the breastfeeding rates were most likely higher among some groups of mothers than others [12].

Several policies have been implemented since the 1960s to address sociodemographic and health inequalities that have historically affected women in GB. These include the enactment of legislation to provide women with equal pay, paid maternity leave and protection from discrimination on the grounds of pregnancy, childbirth and breastfeeding [13]. Advances in women’s reproductive health and rights, including the introduction of oral contraceptives to the UK in 1961 and the passing of the Abortion Act in 1967, may have also contributed to the postponement of childbearing until older age and changes in family formation, including a decline in marriages and an increase in cohabiting and single parenting [14, 15]. In addition, changes in the economic structure of GB since the 1970s coincide with an increasing participation of women in higher education and paid employment outside the home [14]. Over time, these social changes have altered the sociodemographic characteristics of women who become mothers in GB, and may have influenced the magnitude of the inequality in breastfeeding practices between them [16].

To date, no study has investigated the increasing trends in breastfeeding rates in GB between 1985 and 2010, especially the extent to which these trends may have been influenced by sociodemographic changes. Therefore, the aim of this study is to describe the changing sociodemographic characteristics of mothers in GB between 1985 and 2010, and to investigate the extent to which these changes may explain the increasing rate of breastfeeding initiation observed during this period. We have undertaken a similar investigation of trends in breastfeeding continuation and the findings of this will be published soon.

Examining breastfeeding initiation trends, especially inequalities in practice between groups of women, is important for understanding where local and national early interventions to improve breastfeeding practices may have consistently worked less well for some women than others. Such evidence can be used for targeting services in an anticipatory way in the antenatal and early postpartum periods. Since breastfeeding initiation is a prerequisite for breastfeeding continuation and exclusivity, improving the former among all groups of women is fundamental to achieving progress in the latter in GB.

## Methods

### Data

This study used available data from the 1985 to 2010 IFS [5], Briefly, for each year of the IFS, samples of mothers were selected over a 3-month period from all registered live births in England, Wales, Scotland and Northern Ireland. The initial sample sizes ranged from 8,154 mothers in 1985 to 30,760 mothers in 2010, and included an oversample of births from the lowest socioeconomic group. The IFS questionnaires were dispatched by post in three stages and staggered so that mothers received the questionnaire when their infant was 4–6 weeks (stage 1), 4–6 months (stage 2) and 8–10 months (stage 3). All mothers included in the initial sample received the stage 1 questionnaire, while the stage 2 and stage 3 questionnaires were sent to only those mothers who completed the preceding stage. Mothers were reminded by letter or telephone at each stage to complete the questionnaires. The response rate to stage 1 declined from 91% in 1985 to 51% in 2010, while the response rates to stage 2 and stage 3 fluctuated between 80% and 90%, and 81% and 91%, respectively. The data were weighted to correct for differential sampling in each of the four UK countries, the oversampling of births from the lowest socioeconomic group, and attrition at each stage [5].

### Study populations

This present study included mothers who gave birth to a singleton in hospital in England, Wales or Scotland (GB) and who responded to the questionnaires in one of the survey years in 1985 to 2010. Mothers from Northern Ireland were excluded because data for this region was not collected prior to 1990 and ethnicity was not collected. Mothers of multiple births and those who gave birth outside a hospital or maternity unit were also excluded. Few of these mothers responded to the IFS questionnaires and those who responded may have differed from the main study population but were too small a sample to analyse separately. Following these exclusions, the number of mothers included in this present study ranged from 7,262 in 1985 to 12,315 in 2010.

### Study outcome

The main outcome was breastfeeding initiation. This was assessed at stage 1 when infants were 4–10 weeks old, and defined as having ever fed one’s baby breast milk or put him or her to the breast, even if just once.

### Sociodemographic variables

Five maternal sociodemographic variables and one health-related variable (smoking in pregnancy) were defined using available data in each survey year: maternal age at delivery; maternal education (age at which full-time education was completed); socioeconomic status (based on partner’s occupational class under the Standard Occupational Classification in 1985–1990 and mothers’ own occupation groups under the National Statistics Socioeconomic Classification in 2000–2010); partnership status; ethnicity; and smoking in pregnancy. Smoking was included in this present study because it tends to be higher among the more disadvantaged and thus, may be a surrogate measure for poverty [17, 18]. The categories for each of these variables are shown in Table 1.

**Table 1:** Associations between breastfeeding initiation and maternal sociodemographic characteristics, GB, 1985–2010: prevalence and unadjusted odds ratios.

Survey year	1985			1990			1995			2000			2005			2010		
Populations (n^a^)	7,262			7,584			7,047			7,368			9,777			12,315		
Numbers initiated^b^	4,201			4,443			4,424			4,953			7,331			9,821		
% initiated^c^	63.6			63.4			66.5			69.8			76.1			81.4		
	%^c^	OR^d^	95% CI^e^	%	OR	95% CI	%	OR	95% CI	%	OR	95% CI	%	OR	95% CI	%	OR	95% CI
**Maternal age**																		
Under 20	41.4	**0.28**	**(0.23–0.34)**	37.5	**0.19**	**(0.16–0.23)**	43.8	**0.26**	**(0.21–0.32)**	47.2	**0.25**	**(0.20–0.30)**	51.1	**0.20**	**(0.16–0.24)**	58.2	**0.20**	**(0.15–0.25)**
20–24	56.4	**0.51**	**(0.44–0.59)**	52.9	**0.36**	**(0.32–0.40)**	55.6	**0.42**	**(0.36–0.49)**	59.2	**0.40**	**(0.34–0.47)**	67.0	**0.39**	**(0.33–0.45)**	68.9	**0.31**	**(0.27–0.36)**
25–29	69.1	0.88	(0.77–1.02)	65.2	**0.60**	**(0.53–0.67)**	66.9	**0.67**	**(0.59–0.77)**	68.2	**0.59**	**(0.51–0.68)**	76.6	**0.62**	**(0.54–0.72)**	83.8	**0.73**	**(0.64–0.84)**
30 or over	71.6	1.00		75.8	1.00		75.0	1.00		78.4	1.00		84.0	1.00		87.6	1.00	
**Education**																		
16 or under	52.9	**0.37**	**(0.32–0.42)**	50.8	**0.40**	**(0.36–0.46)**	51.7	**0.40**	**(0.35–0.45)**	53.8	**0.46**	**(0.41–0.53)**	59.5	**0.54**	**(0.47–0.61)**	63.5	**0.55**	**(0.48–0.64)**
17 or 18	75.2	1.00		71.8	1.00		72.9	1.00		71.6	1.00		73.3	1.00		75.8	1.00	
Over 18	89.0	**2.66**	**(2.07–3.43)**	91.3	**4.11**	**(3.21–5.27)**	88.5	**2.85**	**(2.32–3.50)**	89.2	**3.28**	**(2.73–3.94)**	91.2	**3.75**	**(3.18–4.42)**	91.6	**3.47**	**(3.01–4.01)**
**Socioeconomic status based on partner’s occupation**																		
Managerial & professional	86.8	**4.23**	**(3.03–5.91)**	87.6	**4.73**	**(3.47–6.44)**	90.0	**5.10**	**(3.61–7.22)**	-	-	-	-	-	-	-	-	-
Intermediate	80.7	**2.69**	**(2.25–3.21)**	79.5	**2.60**	**(2.18–3.10)**	81.6	**2.52**	**(2.11–3.01)**	-	-	-	-	-	-	-	-	-
Skilled-Non-manual	75.9	**2.03**	**(1.61–2.55)**	73.8	**1.89**	**(1.50–2.37)**	71.4	**1.41**	**(1.11–1.80)**	-	-	-	-	-	-	-	-	-
Skilled-Manual	60.9	1.00		59.9	1.00		63.8	1.00		-	-	-	-	-	-	-	-	-
Semi-skilled	57.8	0.88	(0.74–1.04)	53.4	**0.77**	**(0.65–0.91)**	57.6	**0.77**	**(0.63–0.94)**	-	-	-	-	-	-	-	-	-
Unskilled	44.2	**0.51**	**(0.41–0.63)**	41.2	**0.47**	**(0.34–0.64)**	50.5	**0.58**	**(0.44–0.76)**	-	-	-	-	-	-	-	-	-
Unclassified	42.6	**0.48**	**(0.41–0.56)**	48.3	**0.63**	**(0.54–0.72)**	50.5	**0.58**	**(0.50–0.67)**	-	-	-	-	-	-	-	-	-
**Socioeconomic status based on mother’s occupation**																		
Managerial & professional	-	-	-	-	-	-	-	-	-	85.2	**3.92**	**(3.32–4.64)**	88.0	**3.92**	**(3.38–4.56)**	90.7	**3.37**	**(2.89–3.92)**
Intermediate	-	-	-	-	-	-	-	-	-	74.4	**1.97**	**(1.67–2.32)**	77.7	**1.86**	**(1.60–2.17)**	80.3	**1.41**	**(1.20–1.64)**
Routine and manual	-	-	-	-	-	-	-	-	-	59.5	1.00		65.1	1.00		74.4	1.00	
Never worked	-	-	-	-	-	-	-	-	-	52.6	**0.75**	**(0.64–0.89)**	65.9	1.03	(0.83–1.28)	71.5	0.87	(0.71–1.05)
Unclassified	-	-	-	-	-	-	-	-	-	67.1	**1.39**	**(1.13–1.70)**	69.8	1.23	(0.90–1.68)	80.5	**1.43**	**(1.15–1.77)**
**Ethnicity**																		
White	-	-	-	-	-	-	-	-	-	67.9	1.00		73.6	1.00		78.8	1.00	
BME^f^	-	-	-	-	-	-	-	-	-	89.5	**4.02**	**(2.88–5.60)**	93.1	**4.84**	**(3.66–6.41)**	94.5	**4.65**	**(3.54–6.11)**
**Partnership status**																		
Married	68.0	1.00		69.1	1.00		72.8	1.00		77.7	1.00		84.9	1.00		88.8	1.00	
Cohabiting	54.6	**0.57**	**(0.47–0.68)**	53.1	**0.51**	**(0.43–0.59)**	61.2	**0.59**	**(0.51–0.68)**	62.5	**0.48**	**(0.42–0.55)**	70.5	**0.42**	**(0.38–0.48)**	77.2	**0.43**	**(0.37–0.49)**
Single	38.5	**0.29**	**(0.25–0.34)**	42.4	**0.33**	**(0.29–0.38)**	46.0	**0.32**	**(0.28–0.37)**	50.5	**0.29**	**(0.25–0.34)**	56.0	**0.23**	**(0.20–0.26)**	61.9	**0.20**	**(0.18–0.24)**
**Smoking in pregnancy**																		
Yes	47.6	**0.36**	**(0.32–0.41)**	47.5	**0.38**	**(0.34–0.43)**	47.1	**0.33**	**(0.29–0.37)**	49.7	**0.31**	**(0.27–0.36)**	53.6	**0.25**	**(0.23–0.28)**	59.5	**0.25**	**(0.22–0.29)**
No	68.2	0.86	(0.72–1.03)	66.3	**0.82**	**(0.69–0.98)**	69.4	**0.83**	**(0.70–0.99)**	62.8	**0.54**	**(0.40–0.71)**	60.2	**0.33**	**(0.24–0.45)**	63.2	**0.29**	**(0.22–0.39)**
Non-smoker	71.4	1.00		70.5	1.00		73.2	1.00		76.0	1.00		82.1	1.00		85.5	1.00	

^a^n = unweighted population of mothers analysed for BF outcome in each survey.

^b^Numbers who initiated breastfeeding in each survey year are unweighted.

^c^% = weighted proportion who initiated breastfeeding in each survey year.

^d^OR = odds ratio and is unadjusted for other variables.

^e^CI = confidence interval.

^f^BME = black and minority ethnicity.

Bold figures = statistically significant.

p = <0.10 unless indicated by asterisks.

Dash (–) indicates that the variable was not assessed by the IFS in that survey year.

### Statistical analyses

For each survey year, the sociodemographic characteristics (including smoking in pregnancy) were summarised using the weighted proportions of mothers in each category of the variable. A test for linear trend was used to estimate whether the proportion of mothers in each category showed a statistically significant change (p<0.05) from 1985 to 2010. Multivariable logistic regression was used to estimate the independent associations between having initiated breastfeeding and each variable in any given survey year. Associations were adjusted for maternal sociodemographic characteristics; pregnancy-related factors including baby’s birthweight, type of delivery, and length of hospital stay; and support factors including antenatal infant feeding counselling, professional and informal support with infant feeding immediately after giving birth, skin-to-skin contact between mother and baby, and rooming in (whether baby stayed beside mother) during hospital stay. Finally, a test for statistical heterogeneity was performed to ascertain whether the association between each variable and breastfeeding initiation varied over time. Random-effects meta-analysis models were used under the assumption that there was some variability between the survey years [19]. A p value less than 0.10 suggested the presence of statistically significant variability [20]. All proportions and odds ratios (OR) were weighted to account for design effects and attrition using survey commands in Stata version 13.1 [21]. The IFS had ethical approval, and this study did not require further approvals as all data were anonymised and freely accessible to researchers from the UK Data Archive.

## Results

### Changes in maternal sociodemographic characteristics between 1985 and 2010

**Fig 1:**
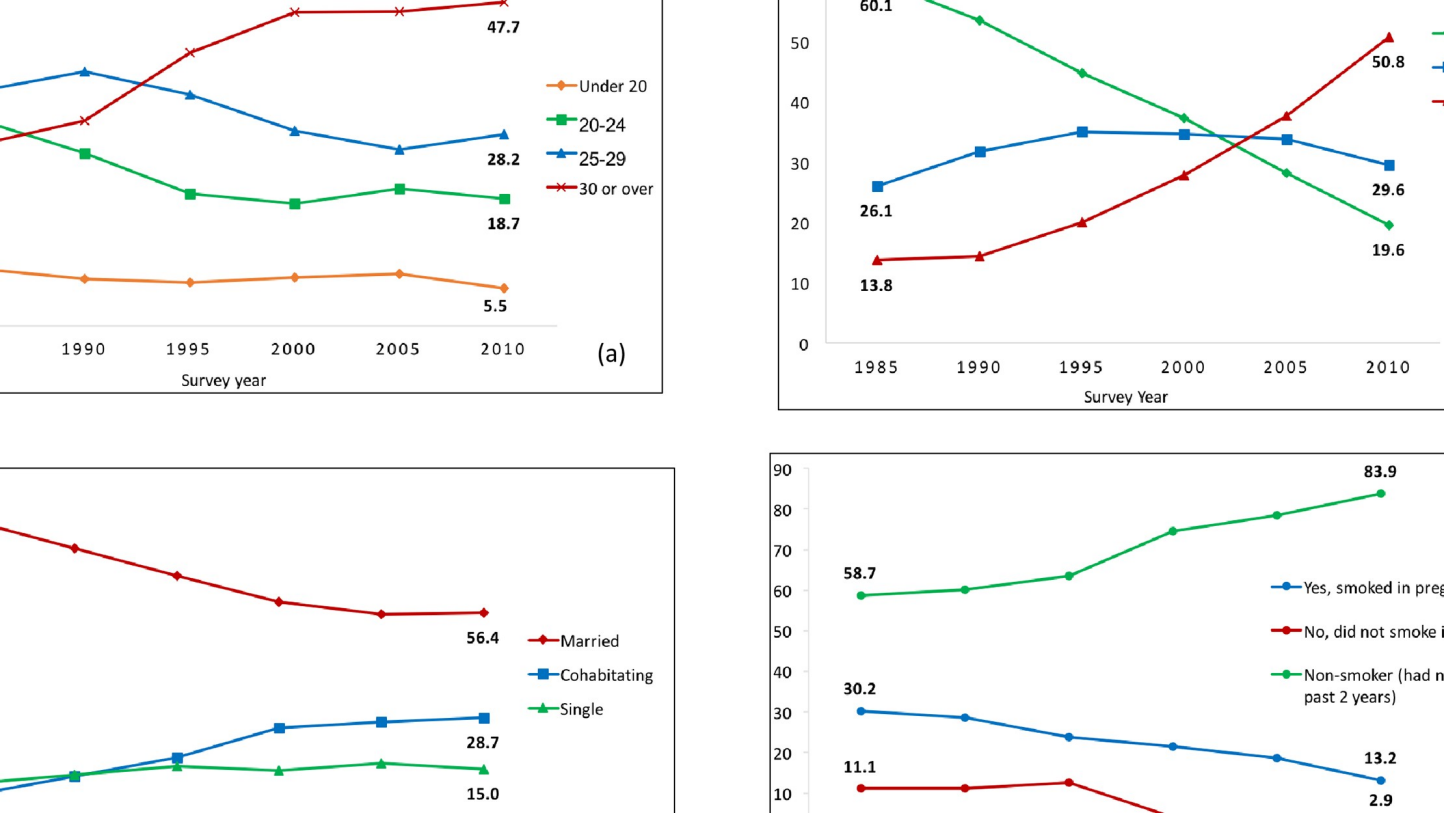
Changing distributions of the sociodemographic characteristics of mothers in GB, 1985 to 2010. A) Proportion of mothers by age at delivery (maternal age); B) Proportion of mothers by education; C) Proportion of mothers by partnership status; D) Proportion of mothers by smoking status in pregnancy.

The proportion of mothers who participated in higher education increased dramatically from 14% in 1985 to 51% in 2010 (Fig 1). There was a trend toward delayed childbearing and a corresponding increase in older maternal age. Cohabitation and, to a lesser extent, single parenting increasingly replaced marriage, and smoking in pregnancy became less common (Fig 1).

There was also an increase in the proportion of mothers who self-identified as being of BME origin compared to being white, rising from 6.9% in 2000 to 14.1% in 2010 (linear trend p<0.001). Finally, there was an increase in the proportion of mothers in the managerial and professional socioeconomic group, rising from 5.8% in 1985 to 6.7% in 1995 (based on partner’s occupation; linear trend p = 0.08), and from 29.2% in 2000 to 34.6% in 2010 (based on mothers’ own occupation; linear trend p<0.001). About 20% to 30% of mothers were assigned to the skilled-manual group in 1985 to 1995 based on their partner’s occupation; although by 1995, a similar proportion of mothers were also assigned to the intermediate group, which includes clerical, service and technical occupations. A disproportionately higher percentage of mothers were unclassified in 1985 to 1995 compared to 2000 to 2010, which may be due to the inclusion of single mothers and those who did not register a partner in the ‘unclassified’ socioeconomic group in 1985 to 1995.

### Sociodemographic inequalities in breastfeeding initiation from 1985 to 2010

#### Maternal age

**Fig 2:**
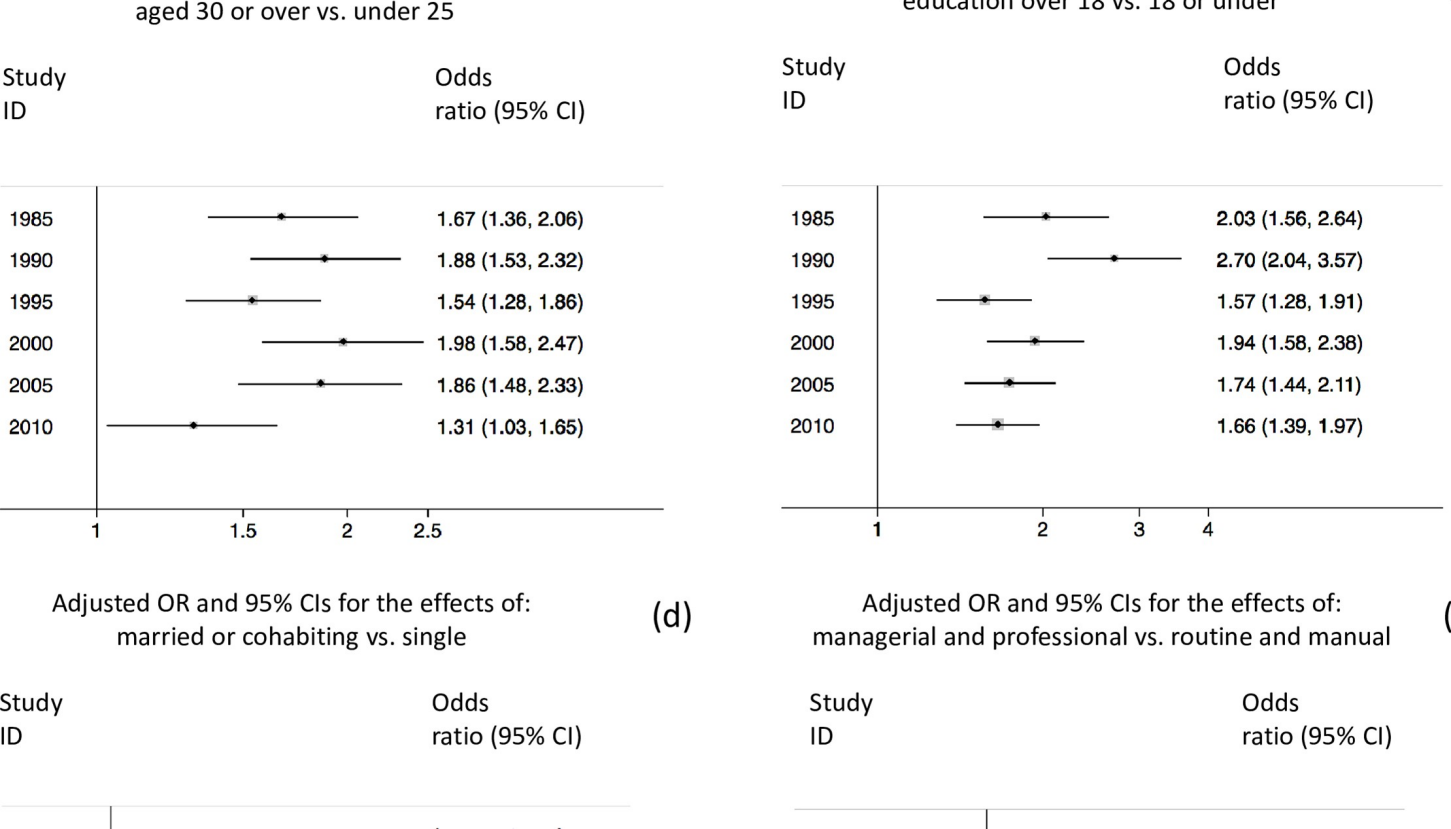
Changes in the associations between breastfeeding initiation and sociodemographic subgroups of mothers in GB, 1985 to 2010. A) Maternal age; B) Education; C) Ethnicity; D) Partnership status; E) SES; F) Smoking in pregnancy.

In unadjusted analyses, there was a positive graded association between maternal age and breastfeeding initiation, with mothers who were aged 30 or over being most likely to initiate breastfeeding in each survey year (p<0.10) (Table 1). Accounting for other factors attenuated this association. However, in general, mothers aged 30 or over remained more likely to initiate breastfeeding than mothers under 30 (p<0.05). (Table 2). There was no significant heterogeneity in this association over time (p = 0.11), with the adjusted OR (aOR) ranging between 1.31 and 1.98 (Fig 2A).

**Table 2:** Associations between breastfeeding initiation and maternal sociodemographic characteristics, GB, 1985–2010: adjusted odds ratios.

Survey year	1985		1990		1995		2000		2005		2010	
Populations (n)	7,262		7,584		7,047		7,368		9,777		12,315	
Numbers initiated	4,201		4,443		4,424		4,953		7,331		9,821	
% initiated	63.6		63.4		66.5		69.8		76.1		81.4	
	OR^a^	95% CI	OR	95% CI	OR	95% CI	OR	95% CI	OR	95% CI	OR	95% CI
**Maternal age**												
Under 20	**0.36**	**(0.27–0.49)**	**0.34**	**(0.25–0.46)**	**0.43**	**(0.32–0.58)**	**0.40**	**(0.29–0.54)**	**0.38**	**(0.28–0.52)**	0.75	(0.50–1.13)
20–24	**0.64**	**(0.52–0.79)**	**0.57**	**(0.46–0.70)**	**0.74**	**(0.59–0.92)**	**0.54**	**(0.43–0.68)**	**0.59**	**(0.47–0.75)**	0.79	(0.62–1.00)
25–29	0.85	(0.70–1.04)	**0.74**	**(0.61–0.90)**	1.05	(0.87–1.26)	**0.75**	**(0.62–0.90)**	**0.65**	**(0.53–0.79)**	1.02	(0.83–1.24)
30 or over	1.00		1.00		1.00		1.00		1.00		1.00	
**Education**												
16 or under	**0.57**	**(0.49–0.67)**	**0.67**	**(0.57–0.78)**	**0.66**	**(0.56–0.78)**	**0.72**	**(0.61–0.85)**	0.93	(0.77–1.12)	1.00	(0.81–1.23)
17 or 18	1.00		1.00		1.00		1.00		1.00		1.00	
Over 18	**1.43**	**(1.08–1.90)**	**2.13**	**(1.59–2.86)**	**1.28**	**(1.01–1.64)**	**1.65**	**(1.32–2.05)**	**1.69**	**(1.38–2.08)**	**1.66**	**(1.37–2.00)**
**Socioeconomic status based on partner’s occupation**												
Managerial & professional	**1.65**	**(1.13–2.41)**	**1.62**	**(1.13–2.32)**	**2.01**	**(1.30–3.13)**	-	-	-	-	-	-
Intermediate	**1.50**	**(1.21–1.87)**	**1.32**	**(1.06–1.64)**	**1.42**	**(1.13–1.79)**	-	-	-	-	-	-
Skilled-Non-manual	**1.50**	**(1.16–1.95)**	1.06	(0.78–1.44)	0.95	(0.71–1.28)	-	-	-	-	-	-
Skilled-Manual	1.00		1.00		1.00		-	-	-	-	-	-
Semi-skilled	1.02	(0.82–1.25)	0.91	(0.73–1.13)	0.89	(0.68–1.16)	-	-	-	-	-	-
Unskilled	**0.67**	**(0.51–0.90)**	**0.62**	**(0.40–0.94)**	0.84	(0.60–1.17)	-	-	-	-	-	-
Unclassified	0.94	(0.67–1.33)	1.06	(0.80–1.41)	**0.77**	**(0.63–0.95)**	-	-	-	-	-	-
**Socioeconomic status based on mother’s occupation**												
Managerial & professional	-	-	-	-	-	-	**1.28**	**(1.02–1.60)**	1.16	(0.95–1.42)	1.13	(0.91–1.41)
Intermediate	-	-	-	-	-	-	0.98	(0.80–1.21)	1.00	(0.82–1.23)	0.87	(0.69–1.08)
Routine and manual	-	-	-	-	-	-	1.00		1.00	1.00	1.00	
Never worked	-	-	-	-	-	-	0.97	(0.76–1.25)	0.79	(0.58–1.09)	**0.70**	**(0.50–0.96)**
Unclassified	-	-	-	-	-	-	0.97	(0.73–1.28)	0.77	(0.47–1.25)	0.99	(0.73–1.34)
**Ethnicity**												
White	-	-	-	-	-	-	1.00		1.00		1.00	
BME	-	-	-	-	-	-	**2.75**	**(1.74–4.33)**	**3.25**	**(2.18–4.86)**	**3.17**	**(2.10–4.78)**
**Partnership status**												
Married	1.00		1.00		1.00		1.00		1.00		1.00	
Cohabiting	0.92	(0.72–1.18)	0.89	(0.72–1.10)	1.01	(0.82–1.24)	0.89	(0.73–1.07)	0.92	(0.75–1.12)	**0.70**	**(0.58–0.84)**
Single	**0.51**	**(0.35–0.74)**	**0.62**	**(0.45–0.85)**	**0.71**	**(0.52–0.97)**	**0.71**	**(0.56–0.90)**	**0.67**	**(0.53–0.86)**	**0.42**	**(0.33–0.53)**
**Smoking in pregnancy**												
Yes	**0.66**	**(0.57–0.77)**	0. 88	(0.75–1.04)	**0.70**	**(0.59–0.83)**	0.86	(0.71–1.04)	**0.71**	**(0.58–0.87)**	0.89	(0.71–1.12)
No	1.09	(0.87–1.36)	1.12	(0.89–1.41)	1.06	(0.85–1.32)	0.87	(0.57–1.33)	0.66	(0.44–1.00)	**0.66**	**(0.45–0.97)**
Non-smoker	1.00		1.00		1.00		1.00		1.00		1.00	

^a^OR = odds ratio and is adjusted for maternal sociodemographic variables; pregnancy-related variables including baby’s birthweight, type of delivery and length of hospital stay; and support factors including antenatal infant feeding counselling, professional and informal support with infant feeding immediately after giving birth, skin-to-skin contact between mother and baby, and rooming in (whether baby stayed beside mother) during hospital stay.

Bold figures = statistically significant

p = <0.05 unless indicated by asterisks

#### Education

Mothers with higher education (over age 18) were more likely to initiate breastfeeding than those who completed full-time education at age 17 or 18 (p<0.10) (Table 1). Even after adjustments, this association remained in each year: In 1985, the aOR was 1.43 (95% CI: 1.08–1.90) and in 2010, the aOR was 1.66 (95% CI: 1.37–2.00) (Table 2). This association also remained when comparing mothers who were over 18 with those who were 18 or under when they completed full-time education, with aORs ranging from 1.66 and 2.70 (Fig 2B). It further indicates that there was heterogeneity in this association over time (p = 0.03), largely due to the widening disparity in favour of mothers with higher education in 1990.

#### Socioeconomic status

In unadjusted analyses, mothers in the managerial and professional socioeconomic group were over twice as likely to initiate breastfeeding than mothers in the routine and skilled-manual groups in each survey year (p<0.10) (Table 1). Adjustments for other factors attenuated this association such that socioeconomic status was no longer associated with breastfeeding initiation in 2000 and 2005. Nevertheless, mothers in the managerial and professional socioeconomic group remained significantly more likely to initiate breastfeeding than mothers in the routine and skilled-manual groups in 1985 (aOR = 1.65), 1990 (aOR = 1.62), and 1995 (aOR = 2.01) (Table 2). Fig 2C shows that there was considerable heterogeneity in this association over the period 1985 to 2010 (p<0.01).

#### Partnership status

Mothers who were married showed higher odds of initiating breastfeeding in each survey year than mothers who were cohabiting with a partner or single (p<0.10) (Table 1). After adjusting for other factors, this association persisted: single mothers remained significantly less likely than married mothers to initiate in each year except 1995; however cohabiting mothers were significantly less likely to initiate than married mothers in 2010 only (aOR: 0.70, 95% CI: 0.58–0.84), with no difference between these group in 1985 to 2005 (Table 2). Mothers who had a partner (whether married or cohabiting) were more likely to initiate breastfeeding than single mothers in each survey year (Fig 2D). Over time, this disparity showed a decrease from 1990 to 2000, followed by an increase from 2005 to 2010 (p = 0.08).

#### Ethnicity

Mothers who were of BME origin were significantly more likely to initiate breastfeeding than white mothers, even after accounting for other factors, with aORs approximately equal to 3 in 2000- to 2010 (Table 2). There was no statistically significant variability in this association over time (p = 0.85) (Fig 2E).

#### Smoking status during pregnancy

Unadjusted analyses indicate that mothers who smoked in pregnancy were significantly less likely to initiate breastfeeding in each survey year than non-smokers (p<0.10) (Table 1). After accounting for other factors, mothers who smoked in pregnancy remained significantly less likely to initiate breastfeeding than non-smokers in 1985 (aOR: 0.66), 1995 (aOR: 0.70), and 2005 only (aOR: 0.71) (Table 2). There was some variability in this association between survey years (p = 0.04) (Fig 2F).

## Discussion

Overall the rate of breastfeeding initiation increased steadily from 1985 to 2010. However, the findings of this present study suggest that sociodemographic inequalities in the practise of breastfeeding initiation persisted between groups of mothers in each survey year, and the increasing trend may have been driven by changing sociodemographic characteristics rather than a universal improvement in breastfeeding. Mothers were significantly more likely to initiate breastfeeding in most survey years if they were aged 30 or over compared to under 30; completed higher education compared to high school only; were married or cohabiting with a partner compared to being single; were of BME origin compared to being white; were non-smokers or had not smoked in pregnancy; or were from the managerial and professional socioeconomic group compared to the routine and manual occupational group. These inequalities have been reported by previous studies.

For instance, other studies found that mothers who are older and have higher education are more likely to initiate breastfeeding as they may have more theoretical knowledge about breastfeeding including its health benefits [9, 22–25]. Mothers from higher socioeconomic groups, such as managerial and professional groups, may be more likely to have the practical means to breastfeed due to having higher-skilled occupations with higher incomes and greater access to maternity entitlements and family-friendly working conditions [9]. In this present study, the disparity in initiation between socioeconomic groups was statistically significant in 1985, 1990 and 1995 only, when mothers’ socioeconomic status was based on their partner’s occupation. This concurs with previous studies in which maternal education accounted for the association between breastfeeding and socioeconomic status based on maternal income or occupation [9, 26, 27].

As with mothers, the higher occupation groups of partners may be indicative of their higher income and potential earnings, which may augment or help to replace women’s wages in the short term after childbirth and encourage women to take time out of work for child rearing. Partners may also influence breastfeeding initiation through their own knowledge and attitudes about breastfeeding, and their provision of practical support including their level of involvement in childcare and household labour [28–32].

The strong positive effect of BME origin on breastfeeding initiation in this present study is also consistent with previous studies. It provides further compelling evidence of the strong influence of social context on breastfeeding, and that breastfeeding may be significantly less normalised among white mothers than those of BME origin in GB [6, 10, 30, 33–36].

Lastly, previous studies suggest that mothers are more likely to smoke during pregnancy if they are younger, less well-educated, unmarried, and in lower socioeconomic groups–characteristics which potentially approximate higher poverty levels [17, 18]. However, after adjusting for other sociodemographic differences, smoking during pregnancy was still significantly associated with a lower likelihood of initiating breastfeeding in this present study. Other potential mechanisms for this negative effect may include a deleterious effect of nicotine on breast milk production and new-born suckling reflexes [37], although the evidence in support of these is limited [38].

There was a narrowing in the inequality in breastfeeding initiation between mothers with a husband or partner and single mothers from 1990 to 2000 suggesting that, for a short-lived period, single mothers initiated breastfeeding at a rate closer to that of mothers who had partner support. Of course, other environmental factors not accounted for in this study may have influenced this narrowing and the reversion that followed for the rest of the study period until 2010. Aside from this variability, any significant heterogeneity that was observed in the associations between breastfeeding initiation and other sociodemographic characteristics most likely resulted from methodological limitations or artefactual effects. Most notably, the variability in the inequality between mothers in the managerial and professional socioeconomic group and those in the routine and manual group was most likely a reflection of basing maternal socioeconomic status on partner’s occupation in 1985 to 1995, and then on mothers’ own occupation in 2000 to 2010. Based on these findings, there is no significant evidence of a consistent change in the sociodemographic inequalities in the practise of breastfeeding initiation between groups of mothers. Furthermore, there was no change in inequalities that might account for the steady increase in the rate of breastfeeding initiation over the period 1985 to 2010.

It is more likely that the increasing initiation rate resulted from the changes in the sociodemographic characteristics of the childbearing population over time, specifically the considerable increase in the proportion of those mothers who are consistently more likely to breastfeed. These include older mothers, the proportion of whom rose from 27% to 48%; more highly educated mothers, rising from 14% to 51%; cohabiting mothers (11% to 29%); managerial and professional mothers (29% to 35%); and mothers of BME origin (7% to 14%). Trend studies in other high-income countries, such as the United States, Norway and Spain, also found that increasing trends in breastfeeding rates, especially after the 1970s, were driven largely by the increasing prevalence of similar groups of mothers [16, 39, 40]. These dramatic sociodemographic changes were brought about in large part by general social and policy changes aimed at decreasing women’s social and health inequalities.

In the 25 years between 1985 and 2010, successive reforms to employment policies in GB reinforced the protection of women in paid employment from discrimination on the grounds of pregnancy, childbirth and breastfeeding; increased maternity payment rates; extended the coverage and duration of maternity leave; and provided paid paternity and parental leave [41, 42]. Regulations were also introduced to protect part-time workers, the majority of whom are women, from being treated less favourably than full-time workers [43]. One major correlate of the expansion of employment policies was the restructuring of the British economy from male-dominated, labour-intensive, manufacturing jobs to knowledge- and service-based jobs [44]. It has also been postulated that training professionals for the future development of GB’s knowledge- and service-based economy has increasingly been the goal of higher education [45]. Consequently, the rate of participation in higher education in GB has increased rapidly since the late 1980s, most noticeably among women. These reforms undoubtedly contributed to women’s increasing access to more employment opportunities outside the home, especially higher-skilled occupations; their increasing socioeconomic position; and the possibility of successful re-entry into their careers following child rearing [46].

Women’s increasing access to higher education and the availability of more attractive employment options in GB may have also encouraged the increasing immigration of non-European young women and families. The increasing incentive to pursue higher education and paid work, along with landmark policy changes regarding women’s sexual and reproductive rights, shifting societal attitudes towards premarital sex and courtship, and declining expectations that women should marry because of pregnancy or motherhood [15], all may have contributed to changes in family formation in GB [14, 15]. This includes a postponement of childbearing to older age and a considerable decline in marriage in favour of cohabitation [47, 48].

These changes in the sociodemographic characteristics of women in GB also occurred against a background of improvements in national infant feeding policies and strategies, beginning in the 1980s. These include the Baby-friendly Hospital Initiative (BFHI), which enhances breastfeeding support to mothers in hospitals and maternity centres. In the statistical analyses, we adjusted for variables in each survey year (where IFS data was available) that represent elements of these policies and strategies. Although not reported in this present study, the analyses showed that in most survey years, the independent effects of these policies and strategies on breastfeeding initiation were not statistically significant after accounting for other factors including maternal sociodemographic differences. We might expect the effects of sociodemographic factors on breastfeeding initiation to change over time in GB based on the compelling evidence from other high-income countries of a reversal in the effects of sociodemographic factors on breastfeeding practices since the late 1970s [16, 39]. We would not expect the effects of supportive infant feeding policies and strategies like the BFHI to change over time and contribute to a consistent increasing trend in breastfeeding initiation.

### Strengths and limitations

This study is the first investigation into the increasing trends in breastfeeding initiation in GB between 1985 and 2010. To our knowledge, it is also the first study in GB, and one of few studies globally, to examine the extent to which increasing breastfeeding trends may have been influenced by sociodemographic changes among the childbearing population. The study populations were sufficiently large to allow comparisons of prevalence and odds ratios over time. The study populations were also highly representative of the general population of mothers, with similar distributions of the sociodemographic characteristics to those shown by national data on all mothers who gave birth in each of the survey years [49]. Therefore, the findings in this study most likely reflect the sociodemographic trends and inequalities in breastfeeding initiation in the wider childbearing population in GB.

There are some caveats to the study findings. Information about breastfeeding and the sociodemographic and smoking variables were based on maternal self-reports. Therefore, the study results are potentially limited by reporting bias. The associations observed in this study may also be confounded by other unaccounted-for factors. The observational nature of the data also precludes any direct inference of a causal relationship between breastfeeding initiation and the study variables.

Distinction was made between two ethnic groups only, ‘white’ and ‘BME’, because disaggregation of the non-white groups resulted in small population sizes and low power to detect significant associations. It is plausible to treat ethnicity as a binary variable given the similarly wide normalisation of breastfeeding among BME groups compared to white groups. At the same time, it is acknowledged that BME groups are distinct from each other in terms of culture, language, religion, disease profiles, and migration histories, and such diversity may also influence breastfeeding behaviours and the commissioning of relevant services.

There was a substantial decline in the response rate to the IFS from 91% in 1985 to 51% in 2010. Each survey year included survey weights designed by the IFS team that corrected for differential non-response at stage 1 based on maternal sociodemographic and pregnancy-related factors. Therefore, this minimised the risk of selection bias resulting from maternal sociodemographic differences. It is more likely that the declining response rate resulted in part from other factors such as a general exhaustion or indifference (‘survey fatigue’) among respondents toward taking part in research surveys, which may have increased in the general childbearing population over time and affected the response to the stage 1 questionnaire in later surveys.

Finally, this study used the most recently available national data on infant feeding practices in GB. However, the study focused on trends over time and therefore includes data from surveys that are decades old. As the most recent survey was conducted in 2010, we cannot claim that the findings represent the current breastfeeding situation in GB.

## Conclusion

The findings from this study indicate a persistence of sociodemographic inequalities in breastfeeding initiation between groups of mothers in GB from 1985 to 2010. These inequalities were hidden among the increasing rate of breastfeeding initiation at the population level. This increasing rate was most likely driven by the increasing prevalence in the childbearing population of those groups of mother who are consistently most likely to breastfeed. Consequently, the needs of mothers who are least likely to initiate breastfeeding–younger mothers, those with less education, those from lower socioeconomic groups and those of white ethnicity–may have gone unmet by general population-based approaches to support breastfeeding. There remains a need for more targeted interventions to bolster the breastfeeding knowledge, skills, and emotional and practical support for the groups of mothers with unmet needs. Such prioritisation may contribute to increases in GB’s relatively low breastfeeding rates through the universal improvement of breastfeeding practices.
