# Correction: Twenty-five-year trends in breastfeeding initiation: The effects of sociodemographic changes in Great Britain, 1985-2010

**DOI:** 10.1371/journal.pone.0212301

**Published:** 2019-02-07

**Authors:** The PLOS ONE Staff

Figs 1 and 2 are incorrect. The authors have provided corrected versions here. The publisher apologizes for the error.

**Fig 1:**
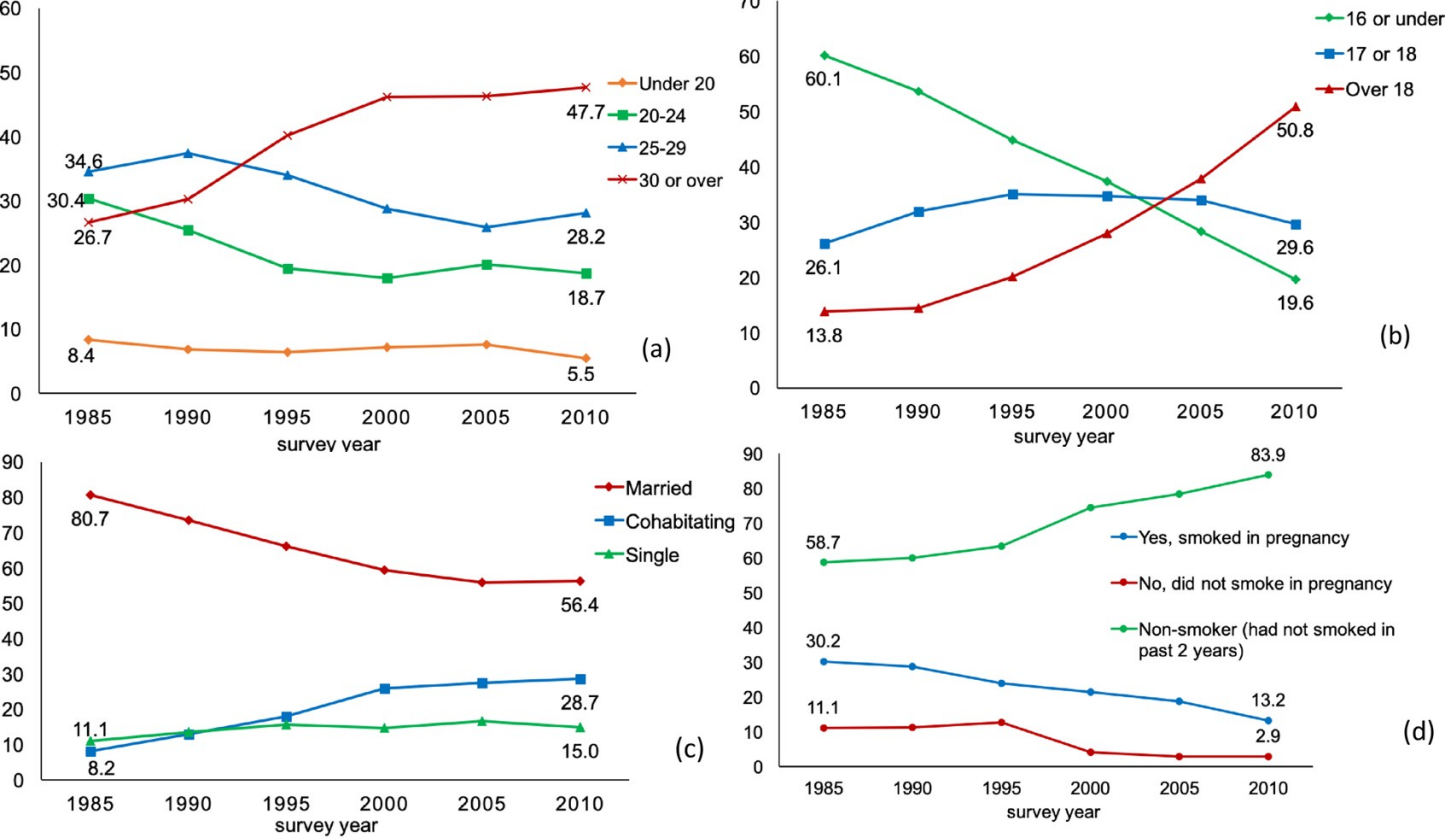
Changing distributions of the sociodemographic characteristics of mothers in GB, 1985 to 2010. A) Proportion of mothers by age at delivery (maternal age); B) Proportion of mothers by education; C) Proportion of mothers by partnership status; D) Proportion of mothers by smoking status in pregnancy.

**Fig 2:**
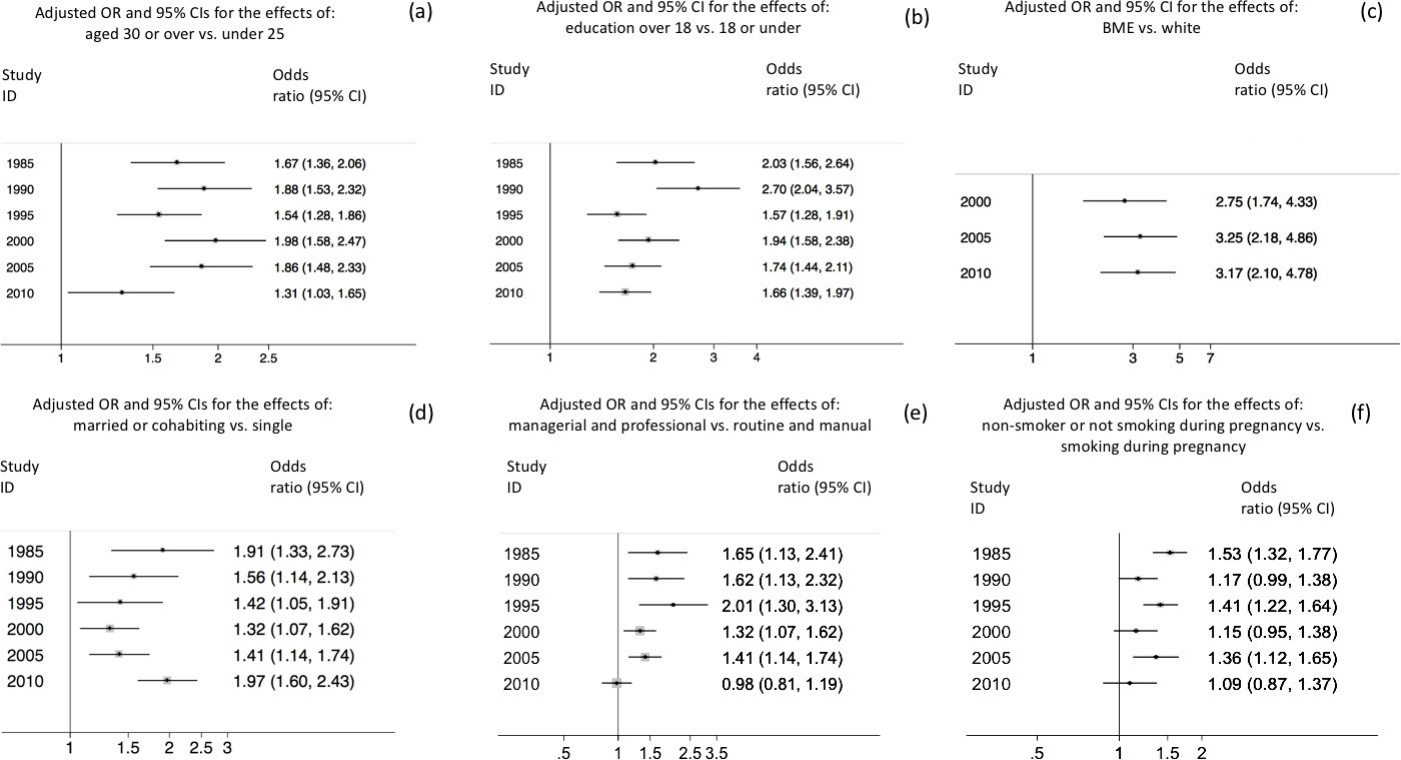
Changes in the associations between breastfeeding initiation and sociodemographic subgroups of mothers in GB, 1985 to 2010. A) Maternal age; B) Education; C) Ethnicity; D) Partnership status; E) SES; F) Smoking in pregnancy.
